# *Brucella* Spondylitis: Current Knowledge and Recent Advances

**DOI:** 10.3390/jcm13020595

**Published:** 2024-01-19

**Authors:** Nikolaos Spernovasilis, Apostolos Karantanas, Ioulia Markaki, Afroditi Konsoula, Zisis Ntontis, Christos Koutserimpas, Kalliopi Alpantaki

**Affiliations:** 1 Department of Infectious Diseases, German Oncology Center, 4108 Limassol, Cyprus; 2 Department of Medical Imaging, University Hospital of Heraklion, 71500 Heraklion, Greece; akarantanas@gmail.com; 3 Advanced Hybrid Imaging Systems, Institute of Computer Science, FORTH, 71500 Heraklion, Greece; 4 Department of Radiology, School of Medicine, University of Crete, 71003 Heraklion, Greece; 5 Internal Medicine Department, Thoracic Diseases General Hospital Sotiria, 11527 Athens, Greece; tzouliamar95@gmail.com; 6 Department of Pediatrics, General Hospital of Sitia, 72300 Sitia, Greece; aphrodite.konsoula@gmail.com; 7 Department of Orthopaedics and Trauma Surgery, Venizeleio General Hospital of Heraklion, 71409 Heraklion, Greece; ntontis1997@gmail.com; 8 Department of Orthopaedics and Traumatology, “251” Hellenic Air Force General Hospital of Athens, 11525 Athens, Greece; chrisku91@hotmail.com

**Keywords:** *Brucella*, brucellosis, spine, spondylitis, spondylodiscitis, imaging

## Abstract

The most prevalent zoonotic disease is brucellosis, which poses a significant threat for worldwide public health. Particularly in endemic areas, spinal involvement is a major source of morbidity and mortality and can complicate the course of the disease. The diagnosis of *Brucella* spondylitis is challenging and should be suspected in the appropriate epidemiological and clinical context, in correlation with microbiological and radiological findings. Treatment depends largely on the affected parts of the body. Available treatment options include antibiotic administration for an adequate period of time and, when appropriate, surgical intervention. In this article, we examined the most recent data on the pathophysiology, clinical manifestation, diagnosis, and management of spinal brucellosis in adults.

## 1. Introduction

Brucellosis is a zoonotic infection caused by the bacterial genus *Brucella*. Humans represent occasional hosts, but brucellosis remains a major public health problem globally and is the most common zoonotic infection. Spinal involvement may complicate the course of the disease and is a significant cause of morbidity and mortality, especially in endemic areas [1].

In this article, we reviewed the current literature on the epidemiology, pathophysiology, clinical presentation, diagnosis, and treatment of a spinal infection due to *Brucella* spp.

## 2. Epidemiology

Brucellosis is caused by a group of small (diameter: 0.5–0.7; length: 0.6–1.5 μm), non-motile, non-spore-forming, slow-growing, facultative intracellular, Gram-negative coccobacilli [2]. It is an ancient disease known by various names, including Mediterranean fever, Malta fever, and undulant fever. The genus *Brucella* was named after David Bruce in 1887. He isolated and identified the causative bacterium from the spleen of a British soldier who had died of a febrile illness that was common among military personnel stationed in Malta [3]. Twelve species are known to date [4], and each has its preferred animal host, although it can also infect other hosts [5]. The major *Brucella* species known to cause disease in humans are *B. melitensis* (sheep and goats), *B. abortus* (cattle, including the vaccine strain RB51), *B. suis* (pigs)*,* and *B. canis* (dogs) [5]. The vast majority of human cases worldwide are associated with *B. melitensis* [6].

The disease can be transmitted to humans through the consumption of unpasteurized animal products (especially raw milk, soft cheese, butter, and ice cream), direct skin or mucous membrane contact with infected animal tissue, or inhalation of infected aerosol particles [6]. The risk of transmission is generally greater for people working with the bacteria in laboratories, slaughterhouses, veterinarians, hunters, shepherds, and meat-packing plant workers. In rare cases, human-to-human transmission has been documented through sexual contact, breastfeeding, congenital transmission, bone marrow transplantation, blood transfusion, and aerosol from an infected patient [7].

Although accurate epidemiologic data are not available for many endemic areas, it is estimated that more than 500,000 new human cases are reported worldwide each year [8]. The disease is most common in people who have travelled to or live in areas where the disease is endemic in animals along the Mediterranean basin (Portugal, Spain, Southern France, Italy, Greece, Turkey, and North Africa), Mexico, South and Central America, Eastern Europe, Asia, Africa, and the Middle East [9,10]. Even though it is a nationally notifiable disease in most countries and must be reported to the local health authorities, this is not always the case, and official numbers represent only a fraction of the actual incidence of the disease [10].

Osteoarticular involvement is one of the most common complications of brucellosis and varies in the literature from 10% to 85% of patients [11,12,13,14,15]. The wide range between reports in the literature may be due to the characteristics of the study populations, the radio-diagnostic methods used, and the different diagnostic criteria [13]. It may present as sacroiliitis, spondylitis, osteomyelitis, peripheral arthritis, bursitis, and tenosynovitis [14]. The type of skeletal involvement depends in part on the age of the patient [1]. The most common osteoarticular finding in children is monoarticular arthritis (usually of the knees and hips) [16], whereas in adults, the sacroiliac (up to 80%) and spinal (up to 54%) joints are most commonly involved [17]. According to one study, patients with osteoarticular brucellosis have a longer duration of illness before diagnosis [11].

*Brucella* spondylitis is among the most serious manifestations of the disease and is associated with complications such as epidural, paravertebral, and psoas abscesses, and possible resultant nerve compression [17]. The incidence of spondylitis among the cases of brucellosis varies in the literature between 2 and 60% [18]. In a review study regarding spinal brucellosis, the predominant radiologic finding was spondylitis or spondylodiscitis, which was documented in 92% of cases, followed by a pre- or paravertebral abscess at a rate of 18% [18]. According to several studies, spondylitis is more common in men and in patients aged between 50 and 60 years [11,19,20]. It mainly affects the lumbar spine, followed by the thoracic, sacral, and cervical areas [21]. The most frequently involved site of infection is the L5–S1 level [15,21]. One study showed that although the lumbar spine is most commonly affected, the involvement of the thoracic spine was more frequent in severely complicated cases [19]. Notably, multilevel vertebral involvement has been reported to occur in 2–36% of cases of *Brucella* spondylitis [11,18,19,20,21,22,23].

## 3. Pathogenesis

Brucellosis may present as a multisystemic disease. Infectious organisms have been described to reach the spine by hematogenous or non-hematogenous routes, such as direct external bacterial inoculation or contiguous spread from an adjacent infectious site [24]. As for *Brucella* species, they mainly spread to the spine hematogenously through the nutrient arterioles of the vertebral bodies [25] or, rarely, by retrograde flow through the venous plexus of Batson, which was first described in an attempt to explain the preference of metastatic disease for the posterior aspect of the vertebral body [26,27]. As the vascularization of the vertebral bodies has been meticulously studied, the natural history of *Brucella* spondylitis can be explained sufficiently. Early *Brucella* spondylitis involves the anterior portion of the vertebral rim as the arterial vascularization of the vertebral bodies is anatomically denser on that surface [28]. Later, the infection progresses to the remainder of the vertebral body using the medullary spaces, eventually reaching the disc annulus and the nucleus pulposus [1,25]. It is worth noting that in adult life intra-osseous arteries are end arteries and therefore, in the event of septic emboli entrapment, extensive destruction of the vertebral body cannot be prevented by the presence of an anastomotic network [29]. The most commonly affected sites are the lumbar spine, followed by the thoracic and cervical spine, while multilevel involvement has also been described [21,30].

Before diving deeper into the pathophysiological mechanisms that orchestrate the deleterious effects of a *Brucella* infection on joints and bones, we will first analyze the key aspects of normal bone physiology. Bone is primarily comprised of cells and an extracellular matrix, the osteoid, which becomes mineralized after the deposition of calcium and phosphate in the form of hydroxyapatite, a process essential for the structural integrity of the bone. There are three types of bone cells: osteoblasts, osteoclasts, and osteocytes [31]. Osteoblasts are bone-forming cells responsible for bone mineralization and the production of the receptor activator of nuclear factor kappa-B ligand (RANKL) and osteoprotegerin, which induce and suppress osteoclastogenesis, respectively [32]. Osteocytes are terminally differentiated osteoblasts that become entrapped in the mineralized matrix [31]. Finally, osteoclasts are bone-resorbing cells with the unique ability to digest the calcified bone matrix. Until recently, it was established that the formation of osteoclasts can be accomplished either by the fusion of osteoclast progenitor cells that originate from the monocyte/macrophage lineage of the bone marrow or through the differentiation of osteal macrophages, which are the bone marrow resident macrophages [33,34]. Nonetheless, the latest research has demonstrated that peripheral blood mononuclear cells can also fuse and become mature multinucleated osteoblasts and that these may significantly contribute to the bone damage seen during inflammatory conditions such as rheumatoid arthritis [35,36]. 

**Figure 1:**
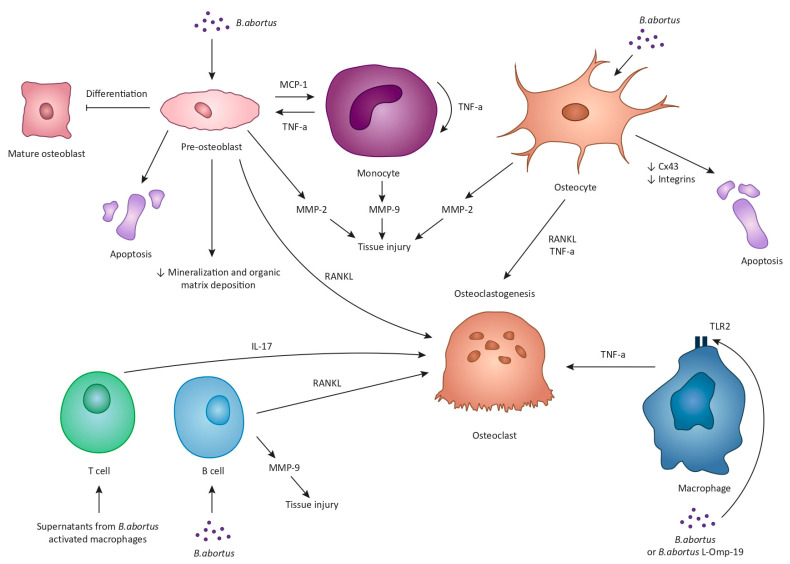
The underlying mechanisms of *Brucella*-induced osteoarticular disease are multiple, complex, and largely rely on experimental data from *B. abortus* studies. *B. abortus* can infect and replicate within osteoblasts and interfere with the physiological functions of these cells via three mechanisms: the induction of osteoblast apoptosis and the hampering of their differentiation; the inhibition of mineralization and organic matrix deposition; and the upregulation of receptor activator of nuclear factor kappa-B ligand (RANKL). *Brucella*-infected osteoblasts also secrete monocyte chemotactic protein 1 (MCP-1) that attracts monocytes and macrophages to the site of infection. In turn, these cells secrete tumor necrosis factor alpha (TNF-a) that, similarly, results in osteoblast apoptosis, decreased bone mineralization, and upregulation of RANKL. *Brucella*-infected osteoblasts and monocytes can also secrete matrix metalloproteinases, MMP-2 and MMP-9, respectively. Specifically, MMP-9 production is the result of the autocrine function of TNF-a produced by monocytes. Additionally, *Brucella* can multiply within osteocytes and lead to the production of MMP-2, RANKL, TNF-a, and proinflammatory cytokines. Moreover, *Brucella* and supernatants from *Brucella*-infected macrophages inhibit the expression of connexin 43 along with the expression of integrins, ultimately leading to osteocyte apoptotic cell death. Upon *Brucella* infection or in response to *B. abortus*, lipidated outer membrane protein 19 macrophages release inflammatory mediators such as TNF-a, eventually enhancing osteoclastogenesis. Moreover, supernatants from *B. abortus*-activated macrophages stimulate T cells to produce interleukin-17 which promotes osteoclast differentiation through the induction of proinflammatory cytokines. Finally, *B. abortus*-infected B cells produce MMP-9 and RANKL.

Bone is often regarded as a metabolically inert structure with an innate resistance to infection. Nevertheless, osteoarticular brucellosis is the most frequent complication of a *Βrucella* infection in humans [11,12]. The underlying mechanisms involved in this process have only recently been elucidated (Figure 1). The available data are mainly derived from research regarding *B. abortus* but can be safely used for the understanding of the pathogenesis of *Brucella* spondylitis in general. By now, it is evident that *Brucella*’s success as a pathogen relies on its ability to maintain an intracellular lifestyle, primarily by invading and replicating within macrophages. However, macrophages are not the only intracellular niche that *Brucella* can penetrate [37]. Firstly, it has been established that *B. abortus* can infect and replicate within osteoblasts in vitro [38,39]. Once inside osteoblasts, *Βrucella* interferes with the physiological functions of these cells via, principally, three mechanisms: the induction of osteoblast apoptosis and the hampering of their differentiation; the inhibition of mineralization and organic matrix deposition; and the upregulation of RANKL [39]. These changes are the result of the direct effect of *Brucella* on osteoblasts, but also the result of *Brucella*-infected macrophages, the ones that already reside in the bone and the ones that are attracted to the site of infection. The induction of apoptosis is largely dependent upon the phosphorylation of p38 and extracellular signal-regulated kinase 1 and 2 (ERK1/2), which is activated in *Brucella*-infected osteoblasts. P38 and ERK1/2 are mitogen-activated protein kinases (MAPK) that regulate a plethora of functions in terms of cell growth, development, and survival [40]. Another critical function of these pathways is the production of monocyte chemotactic protein 1 (MCP-1) by osteoblasts, which is responsible for the attraction of monocytes and macrophages to the site of infection. In turn, these cells secrete tumor necrosis factor alpha (TNF-a) that results in osteoblast apoptosis, decreased bone mineralization, and upregulation of RANKL [39]. 

Matrix metalloproteinases (MMPs) also contribute to the osteoarticular damage in the context of brucellosis. Specifically, two types of MMPs, MMP-2 and MMP-9, which aid in the degradation of type I collagen present in bones and type II collagen present in cartilage, have been demonstrated to be involved in *Brucella*-induced tissue injury [41,42]. In particular, in vitro studies have shown that *B. abortus*-infected osteoblasts produce MMP-2 in a process that is largely mediated by the production of granulocyte-macrophage colony-stimulating factor (GM-CSF) by the same cells [42]. In addition, as mentioned above, *Brucella*-infected osteoblasts produce MCP-1 that attracts monocytes, which then secrete MMP-9 [42]. MMP-9 production is the result of the autocrine function of TNF-a produced by monocytes in response to GM-CSF [42]. 

*Brucella* can also infect and multiply within osteocytes in vitro [43]. Infected osteocytes then secrete MMP-2, RANKL, TNF-a, and proinflammatory cytokines [43]. This response ultimately leads bone marrow-derived monocytes (BMM) to undergo osteoclastogenesis. At this point it should be mentioned that one of the ways by which coordinated communication among osteocytes and between osteocytes and osteoblasts is achieved is via gap junctions, and the most abundant protein in these gap junctions is connexin 43 (Cx43) [44]. Interestingly, the *B. abortus* infection has been found to reduce the expression of Cx43 [43]. Moreover, the interaction between osteocytes and supernatants from *Brucella*-infected macrophages inhibits the expression of Cx43 along with the expression of integrins [43], which also participate in osteocyte adhesion and signaling [45]. The outcome of these changes is osteocyte apoptotic cell death [43]. Based on these findings, it can be safely deducted that *Brucella* harms osteocyte activity and viability, directly and indirectly, thus contributing to the tissue damage observed in an osteoarticular infection. 

The role of macrophages and monocytes in the pathophysiology of tissue damage noted in *Brucella* infections is not limited to their interaction with osteoblasts and osteocytes. Upon infection with *Brucella* or in response to *Brucella* lipoproteins, such as the lipidated outer membrane protein 19 (L-Omp19), macrophages release inflammatory mediators such as TNF-a, interleukin-6 (IL-6), and IL-1β in a toll-like receptor 2-dependent manner (TLR2) [46]. In turn, TNF-a production results in the differentiation of BMM into osteoclasts [46]. Another intriguing observation is that supernatants from *B. abortus*-infected monocytes or L-Omp19-stimulated monocytes are able to induce, again, through TNF-a production, the differentiation of human monocytes to osteoclasts [46]. It should be pointed out that osteoclastogenesis associated with *B. abortus* does not require bacterial viability but is equally elicited by structural bacterial components. It is established that these components are the *Brucella* lipoproteins but not the *Brucella* lipopolysaccharide [46,47]. 

*Brucella* affects the bone tissue not only through macrophages and monocytes but also through T cells and B cells by exploiting them to induce bone loss. Specifically, stimulation of activated T cells with supernatants from *B. abortus*-activated macrophages results in the production of RANKL and IL-17 which promote osteoclastogenesis in vitro [48]. In addition, it appears that IL-17 is the main driving force for osteoclast differentiation through the induction of proinflammatory cytokines, primarily TNF-a, by osteoclast precursors [48]. This phenomenon has also been replicated in vivo when injection of mice tibiae with T cells that were treated with supernatants from *Brucella*-infected macrophages induced extensive osteoclastogenesis [48]. Similarly, *B. abortus*-infected B cells produce MMP-9, proinflammatory cytokines, and RANKL, the latter being the main mediator of B cell-induced osteoclastogenesis in vitro [49].

Finally, the role of several cytokines, their receptors, and single-nucleotide polymorphisms for cytokine-encoded genes in the inflammatory damaged observed during *Brucella* spondylitis is still unclear and demands further research [50,51,52]. 

In summary, the osteoarticular damage observed in a *Brucella* infection is the aftereffect of the direct changes that the bacterium causes on bone cells and also the result of the intricate interactions between *Brucella*, bone cells, and the immune system.

## 4. Clinical Features

Brucellosis in humans affects numerous systems and manifests with a wide range of symptoms, in both acute and chronic forms, but it can also be asymptomatic [53]. Fever, chills, headaches, malaise, and fatigue are some of the most prevalent, nonspecific symptoms of both uncomplicated and complicated forms of the disease. Other symptoms and signs are abdominal pain, splenomegaly, and hepatomegaly [54]. Apart from all the aforementioned symptoms, brucellosis should also be considered in the differential diagnosis of a fever of unknown origin, especially in non-endemic areas [55]. Complicated forms of the disease include osteoarticular, genitourinary, neurologic, cardiovascular, and pulmonary involvement [56,57].

In a recent systematic review, it has been shown that in approximately one third of adult patients, brucellosis manifests as spondylitis or sacroiliitis [58]. In a set of different studies, the percentage of osteoarticular involvement ranged between 20% and 60%, and the percentage of spondylitis between 8% and 13% [59]. When it comes to the musculoskeletal manifestations of the infection, sacroiliitis and hip joint involvement are more common in young individuals in the acute form of the disease, whereas spondylitis and spondylodiscitis are more common in the elderly and in chronic forms of the disease [60]. The most commonly afflicted vertebrae in spondylitis are the lumbar (60%), sacral (19%), and cervical (12%) [21]. Lumbar (60–70%), thoracic (20%), and cervical (6–13%) segments are usually implicated in spondylodiscitis [17]. 

There are two forms of spinal brucellosis: localized and diffuse. In localized involvement, osteomyelitis is restricted to the anterior region of an endplate at the discovertebral junction, while in extensive involvement it affects the whole vertebral endplate or the entire vertebral body [17]. 

Arthralgias are present in the majority of adults affected by brucellosis, and approximately half of them suffer from myalgia and back pain [58]. Nevertheless, axial back pain remains a non-specific clinical sign of spinal brucellosis. As a result, many patients presenting with lower back pain combined with sciatic radiculopathy are misdiagnosed or are diagnosed belatedly [17,61].

Because of its association with epidural, paravertebral, and psoas abscesses, and probable nerve compression, spondylitis is a significant brucellosis complication. These types of abscesses are present in a minority of patients suffering from *Brucella* spondylitis and manifest as episodes of high-grade fever, lower back pain, and inability to bear weight, possibly leading to permanent neurological deficits or even death in cases of delayed or inappropriate treatment [17]. Lastly, it is important to keep in mind that tuberculous spondylodiscitis can greatly resemble spinal brucellosis in terms of clinical presentation. However, it appears that systemic symptoms such as fatigue and fever are more common in a *Brucella* infection, whereas back pain, local tenderness, and spinal complications are observed with a higher frequency in tuberculous spondylodiscitis [62].

## 5. Diagnosis

### 5.1. Microbiological Diagnosis

Because brucellosis in humans presents with nonspecific clinical and laboratory findings, a microbiological analysis is crucial for a definite diagnosis. A *Brucella* infection should be suspected in the appropriate clinical context and with relevant epidemiologic exposure (consumption of unpasteurized dairy products, animal exposure in an endemic area, and/or occupational exposure).

Laboratory findings of brucellosis may include mildly elevated erythrocyte sedimentation rate and liver function enzyme levels, as well as hematologic abnormalities such as anemia, leukopenia or leukocytosis with relative lymphocytosis, and thrombocytopenia [1,63]. Rarely, pancytopenia is observed in patients with a *Brucella* infection and is attributed to hypersplenism, hemophagocytic syndrome, diffuse intravascular coagulation, or immune-mediated cellular destruction [6,64,65,66].

According to the CDC and the Council of State and Territorial Epidemiologists, a definitive diagnosis is established by direct detection of *Brucella* species by a culture from a clinical specimen or indirectly by a fourfold or greater increase in *Brucella* antibody titer between serum specimens from the acute and convalescent phases obtained at least 2 weeks apart [67]. However, a presumptive diagnosis is made by a *Brucella* total antibody titer of at least 1:160 in the serum agglutination test (SAT) or *Brucella* microagglutination test (BMAT) in one or more serum specimens obtained after the onset of symptoms or by detection of *Brucella* DNA in a clinical specimen by a polymerase chain reaction (PCR) [67].

A positive blood culture or a positive culture from other specimens (e.g., bone marrow, bone, synovial fluid, cerebrospinal fluid, urine) is the cornerstone of diagnosis [68]. Brucellosis is characterized by initial bacteremia that is followed by a macrophage invasion resulting in a reduction of blood-circulating bacteria [69]. Therefore, at least two or three separate peripheral blood culture sets should be drawn as soon as the disease is suspected [70]. The sensitivity of blood cultures ranges from 10% to 90% [69]. Because of slow growth in culture media, the physician should inform the microbiology laboratory to extend the incubation period up to 4 weeks, although the new BACTEC system has higher reliability and can detect the bacterium within 5 to 7 days. Because of the high rate of transmission to laboratory personnel, biosafety measures should be taken when isolating this organism [71].

Bone marrow culture is more sensitive than blood and is considered the gold standard for the diagnosis of brucellosis, but the invasiveness of the procedure should be considered [72]. In a study of 50 patients diagnosed with brucellosis, the bone marrow culture was positive in 92% of cases. The bone marrow culture has a shorter time of detection than blood culture does, and its sensitivity is not affected by prior antibiotic use. Individuals with chronic infections are less likely to have a positive culture [73]. If focal disease is suspected, such as in cases of spondylitis, samples should be obtained from the infected area (e.g., bone, joint aspirate, cerebrospinal fluid) [8]. Rapid identification of *Brucella* species recovered from cultures is essential to making a timely diagnosis, avoiding biological risk to laboratory personnel, confirming the presence of the disease in its early stages when antibody titers are negative or low/borderline, distinguishing between wild and vaccine *Brucella* strains, and identifying the source of transmission, since the individual species and their naturally occurring hosts are highly interrelated [68].

In patients with a clinically compatible illness, serologic testing is the most commonly used diagnostic method, especially in endemic areas, because they are inexpensive, user-friendly, and have high negative predictive value [68]. The most common serologic tests for detecting specific antibodies in the serum of infected patients are the SAT and the enzyme-linked immunosorbent assay (ELISA). The Rose Bengal agglutination test (RBT) is a rapid, accurate method in the acute phases of the disease and can be used as a screening tool [74]. Other tests that are most useful in chronic or/and complicated cases are the 2-mercaptoethanol (2-ME) agglutination test, the immunocapture agglutination (BrucellaCapt) test, and the indirect Coombs test [68].

The SAT, which measures total IgM, IgA, and IgG antibodies against smooth lipopolysaccharide (S-LPS), remains the most popular method. Although a single titer is not diagnostic, SAT titers > 1:160 outside endemic areas and >1:320 within endemic areas are considered highly suggestive of an infection [6]. Seroconversion and a fourfold or greater increase in titers measured at least 2 weeks apart indicate a definitive diagnosis [6]. SAT can detect antibodies against *B. abortus*, *B. suis*, and *B. melitensis* but not *B. canis* or the vaccine strain RB51 [6,72]. In a study that included patients with a blood culture-proven *Brucella* infection, the initial titer of SAT was ≥1:320 in 96% of patients [75].

When interpreting positive SAT results, the possibility of cross-reactions of IgM antibodies of *Brucella* with other Gram-negative bacteria such as *Υersinia enterocolitica*, *Escherichia coli* O:116 and O:157, *Moraxella phenylpyruvica*, *Francisella tularensis*, certain *Salmonella* serotypes, and from individuals vaccinated against *Vibrio cholerae* should be considered [71,72]. Early, chronic, or complicated disease is associated with high rates of false-negative antibody titers [68].

ELISA is a sensitive quantitative method for measuring specific IgA, IgM, and IgG anti-*Brucella* antibody titers that allows for a better interpretation of the clinical situation. IgM antibodies are predominant in acute infection but decrease within a few weeks. Low IgM titers may persist for months or years after the initial infection. Relapses are accompanied by transient increases in IgG and IgA antibodies, but not IgM [71]. However, until better standardization is established, ELISA should be used in cases of strong clinical suspicion when SAT is negative to confirm the diagnosis or in chronic, focal, or complicated cases [68].

PCR tests can be performed on serum or any tissue samples, such as bone, and allow for a diagnosis within a few hours with high sensitivity and specificity, but are not a routine diagnostic tool. Caution should be taken when interpreting results, as a false positive result could be due to low bacterial inoculum in frequently exposed healthy individuals in endemic areas, DNA from dead bacteria, or a patient who has recovered [76]. In one study, real-time PCR demonstrated high sensitivity (93.5%) and specificity (100%) in formalin-fixed, paraffin-embedded samples from patients with *Brucella* vertebral osteomyelitis who required surgical treatment for neurologic deficits. In terms of sensitivity, real-time PCR proved to be better than blood culture (35.5%), SAT test (80.6%), and Giemsa stain (51.6%) [77].

Lastly, a special mention must be made regarding the pathological features of *Brucella* spondylitis, although these are not routinely used as a diagnostic tool. Firstly, chronic inflammation along with in-acute-phase chronic inflammation are the most commonly encountered pathological changes of spinal brucellosis [78]. Furthermore, histopathology can potentially aid in the differentiation between brucellar and tuberculous spondylitis through specific findings like caseous necrosis, which is typically identified in tuberculous lesions, and through staining markers like Angiopoietin-like protein 4 [79].

### 5.2. Radiological Diagnosis

**Figure 2:**
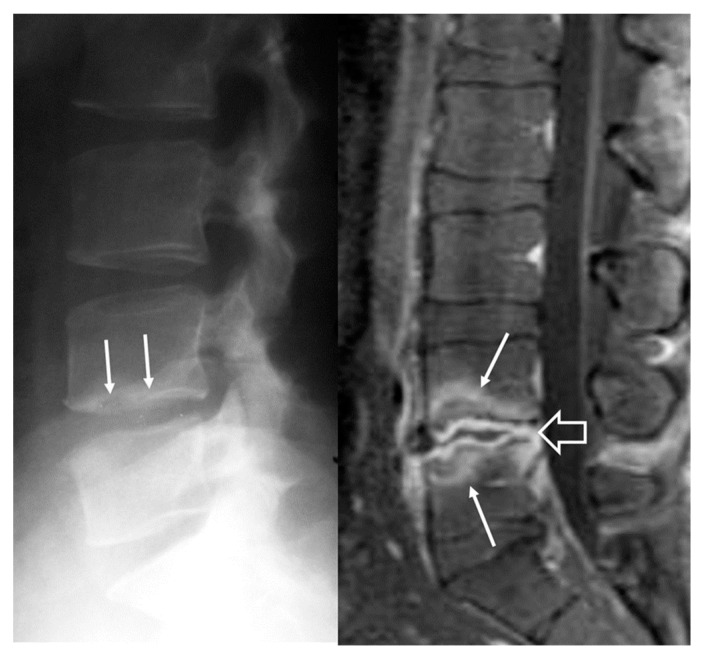
A 34-year-old man with *Brucella* spondylodiscitis. The initial lateral radiograph (**left**) shows a cortical disruption at the inferior epiphyseal plate of L4 vertebral body (arrows). The sagittal fat suppressed contrast enhanced T1-w MR image (**right**) shows septic discitis (open arrow) and bone barrow edema on both L4 and L5 vertebral bodies (arrows), suggesting spondylitis.

The focal form of the disease is confined to the anterior portion of the endplate, typically in the anterior superior of a lumbar vertebra, often at the L4–L5 level [6]. The diffuse form involves the entire vertebral body and extends to the adjacent disc and the paravertebral and epidural space. Multifocal involvement has been described in sporadic cases [80]. Plain radiographs show no findings initially. At about 3–5 weeks after the onset of symptoms, osteolysis demonstrated with loss of the osteosclerotic epiphyseal plate is shown (Figure 2). Focal erosions of the superior or inferior vertebral body are characteristic [30].

A gas vacuum may be observed in the anterior part of the disc, either due to disc ischemia and necrosis or due to focal instability [81]. Osteophytosis at the anterior vertebral endplate is shown in long-standing or poorly treated cases. It has to be pointed out that osseous remodeling may progress slowly, and radiographic findings may simulate degenerative spinal disease. Computed tomography (CT) depicts the changes earlier in the course of the disease, and due to lack of overlapping tissues, gas within the disc can be depicted in 25–30% of the cases. Post-contrast CT may show abscess formation either in the paravertebral spaces or in the spinal canal [82].

**Figure 3:**
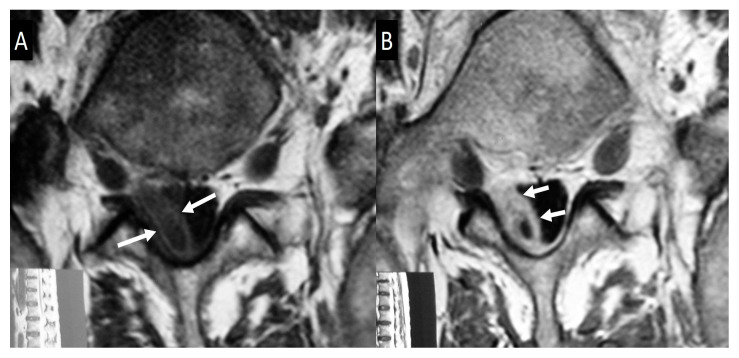
Axial plain (**A**) and contrast-enhanced (**B**) T1-w MR images, showing the epidural abscess formation on the right side (arrows in **A**), with wall enhancement (arrows in **B**), and displacement of the dural sac to the left.

**Figure 4:**
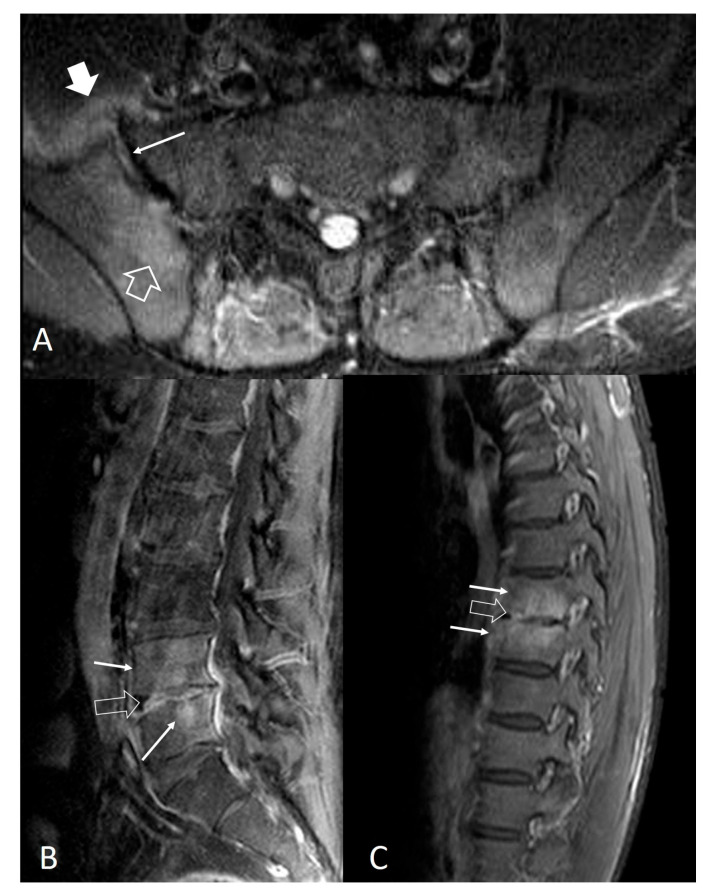
Noncontiguous multifocal musculoskeletal brucellosis. (**A**) Axial STIR MR image, showing bone marrow edema (open arrow), joint effusion (thin arrow), and capsular thickening (thick arrow) in keeping with sacroiliac joint involvement. Sagittal fat-suppressed contrast-enhanced MR images of the lumbar spine (**B**) and thoracic spine (**C**) showing discitis (open arrows) with spondylitis (thin white arrows).

Magnetic resonance imaging (MRI) findings are not specific and follow the typical infection pattern, including a hypointense signal on T1-w images, hyperintensity on T2-w and STIR images, and enhancement of the disc and bone marrow edema foci (Figure 2, Figure 3 and Figure 4) [14,30]. The presence of intracanalicular abscess formation is confirmed with wall enhancement and is an indication for surgical decompression (Figure 3). Similarly, paravertebral abscesses are observed in approximately 30% of cases and are typically demonstrated with wall enhancement [30].

**Figure 5:**
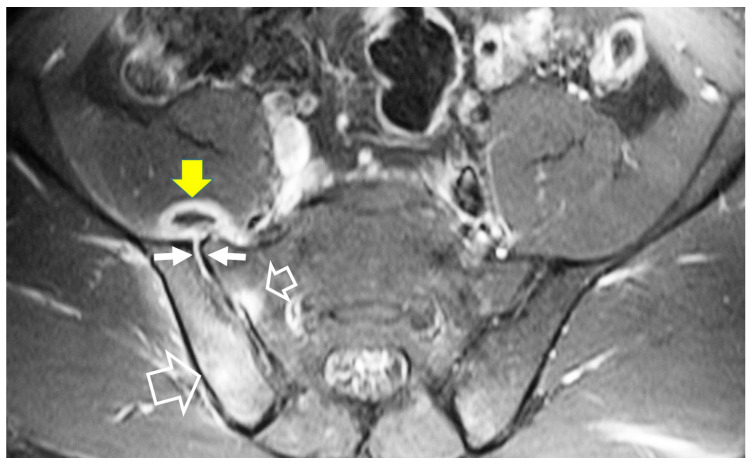
A 23-year-old male with a serologically proven diagnosis of brucellosis 9 months prior to current imaging. The patient received treatment for 3 months and now presents with recurrent symptoms. Axial fat-suppressed T1-w MR image showing enhancing bone marrow edema on both sides of the sacroiliac joints (open arrows), joint effusion (arrows), and anterior capsular thickening and enhancement (yellow arrow) in keeping with septic sacroiliitis.

Extraspinal involvement occurs primarily in the sacroiliac joints and the knee. In most of the cases (>80%), *Brucella* sacroiliitis is unilateral [82]. Radiographic findings of sacroiliitis 3 weeks after the onset of symptoms include disruption of the subchondral sclerotic line and later narrowing or widening of the joint space. Erosions, subchondral sclerosis, and ankylosis of the joint may be seen in chronic cases. Early in the course of the disease, MRI findings are not specific and include bone marrow edema, joint effusion, and capsular thickening (Figure 4 and Figure 5).

The main differential diagnosis of spinal brucellosis is spinal tuberculosis. As a rule, radiographic findings occur later in the course of the disease. In spinal tuberculosis, CT appears to be superior to plain radiographs in identifying endplate irregularity and osseous destruction and can guide a percutaneous biopsy. CT and MRI findings in tuberculosis include contiguous on non-contiguous vertebral involvement with preservation of the disc spaces until later in the course of the disease, prevertebral and paravertebral collections, often in the psoas muscles, with an extension beneath the anterior longitudinal ligament, and epidural abscess formation. A straightforward diagnosis may be difficult in atypical cases, and the differential diagnosis should also be supported by clinical and serological findings [83,84].

## 6. Treatment

### 6.1. Conservative Management

Brucellosis treatment depends largely on the affected parts of the body. Available treatment options include antibiotic administration for an adequate period of time and, when appropriate, surgical intervention. Antibiotics agents which accumulate into phagocytes may be pivotal for the successful treatment of brucellosis. Combinations of tetracyclines, rifampicin, aminoglycosides, trimethoprim-sulfamethoxazole (TMP-SMX), and quinolones have been used [85]. The most commonly used combination regimens in the absence of focal disease are doxycycline (100 mg BID) for 6 weeks plus an aminoglycoside (streptomycin 1 gr OD for 2–3 weeks or gentamicin 5 mg/kg/day OD for 7–10 days) or doxycycline (100 mg bid) plus rifampicin (600–900 mg OD) both for 6 weeks [6,86]. Resistance of *Brucella* species to tetracyclines or aminoglycosides does not occur [87,88], while decreased susceptibility or even resistance to rifampicin has been described [89,90]. Relapses occur usually within the first 6 months of treatment completion and are only rarely due to antibiotic resistance if a combination treatment has been used. Inadequate antimicrobial choice, short treatment duration, undiagnosed focal disease, and lack of compliance are the main reasons for relapse [91]. Most of the relapsed cases respond favorably to a repeated course of the antimicrobial regimen that was administrated during the first episode [92].

While sacroiliitis does not appear to require special treatment, *Brucella* spondylitis requires a longer course of antibiotics than uncomplicated brucellosis, and surgical intervention might be required [85,93,94], while delayed initiation of treatment can result in long-term disability, as is usually the case in spondylodiscitis in general irrespective of the cause [95]. Regrettably, the optimal approach in terms of treatment duration and antibiotic combination has yet to be defined [61,96,97]. A combination of two or three antibiotics is commonly used for 3–6 months and in many cases for even longer [91,98].

In an open, controlled, nonrandomized study which involved only 31 patients with spinal brucellosis treated for a median time of 12 weeks, clinical response did not differ between patients who received ciprofloxacin plus rifampicin and patients who received doxycycline plus streptomycin [99]. In another retrospective observational study there were no significant differences between patients receiving doxycycline-streptomycin and those receiving doxycycline-rifampicin for 3 months but it should be underlined that treatment failure rate ranged between 15–18% [100]. In a large multicenter retrospective comparative study including 293 patients with spinal brucellosis, five major treatment regimens were used for at least 12 weeks: doxycycline plus rifampicin plus streptomycin; doxycycline plus rifampicin plus gentamicin; doxycycline plus rifampicin plus ciprofloxacin; doxycycline plus streptomycin; and doxycycline plus rifampicin [19]. There were no significant differences among these antibiotic groups regarding outcomes [19]. On the contrary, in a recent retrospective cohort study on 100 patients with *Brucella* spondylitis, the triple antibiotic regimen of doxycycline, compound sulfamethoxazole, and rifampicin was more successful in treating *Brucella* spondylitis compared to the dual antibiotic regimen of compound sulfamethoxazole and rifampicin [101].

Many clinicians, including us, favor a triple-regimen antibiotic treatment for *Brucella* spondylitis. The combination of doxycycline (100 mg BID for at least 12 weeks) plus rifampicin (600–900 mg OD for at least 12 weeks) plus streptomycin (1 gr OD for 2–3 weeks) or gentamycin (5 mg/kg/d OD for 5–7 days) is commonly used in adults and is associated with high rates of favorable outcomes and reduced relapse rates [102]. Other treatment regimens are derived from the substitution of the aminoglycoside with a quinolone (e.g., ciprofloxacin 500 mg BID for at least 12 weeks) or TMP-SMX (TMP 10 mg/kg/day and SMX 50 mg/kg/day, both divided in 2 doses for at least 12 weeks) [19,103,104]. Pregnant women can be treated with a combination of two or three of the following antibiotics: rifampicin (600–900 mg OD for at least 12 weeks), TMP-SMX (160 mg TMP/800 mg SMX OD for at least 12 weeks), and ceftriaxone (2 g OD for 4–6 weeks). For pregnant patients ≥36 weeks of gestation, only rifampicin and ceftriaxone are prudent to be administered until delivery, due to the risk of neonatal kernicterus with the use of TMP-SMX in the last 4 weeks of pregnancy [105,106].

### 6.2. Surgical Management

As mentioned, long term administration of antimicrobial agents is the mainstay of treatment of *Brucella* spondylodiscitis [104]. According to Lozano et al., surgery is required in 3% to 29% of patients [107]. Surgical treatment is indicated for patients with neurological symptoms caused by bone deformities and purulent epidural abscesses due to possible irreversible neural damage [108]. Patients with partial or temporary response to antimicrobial therapy, such as patients with large paravertebral abscesses, might also require surgical intervention [107]. There are limited data regarding the surgical treatment of *Brucella* spondylitis. The role of surgical intervention, particularly in patients without neurological symptoms, remains to be determined [108,109]. In the past, the use of spinal implants in the presence of infection was highly controversial. Nowadays, there is sufficient evidence to support the claim that the use of spinal instrumentation in patients with infections is safe since it does not compromise the eradication of the pathogen [110].

#### 6.2.1. Open Surgery

In patients with spinal instability, symptomatic neural compression, or progressive kyphotic deformity, decompression of the spinal canal and stabilization are mandatory. The main surgical approaches include anterior debridement, traditional posterior decompressive procedures with or without instrumentation surgery, and combined anterior and posterior approaches. However, there is no consensus on the optimal surgical approach [111,112]. The surgical approach should be chosen according to the location of the spinal lesion, the degree of vertebral destruction and nerve compression, and the surgeon’s experience and technical skills [113].

In the past, anterior decompression combined with posterior internal fixation was commonly used. The current development of spinal surgery implants and techniques has facilitated the treatment of lumbar brucellosis with abscesses only by the posterior approach [114]. Posterior surgery is considered suitable for intraspinal granulation and abscess removal, especially for patients with nerve compression caused by posterior column lesions, whereas combined surgery is recommended for patients with perivertebral abscess, psoas abscess, or severe anterior column destruction [113,114].

Anterior standalone approach with reconstruction of the spinal column has been described by several authors as safe and effective in the treatment of spinal infections, including cases of *Brucella* spondylitis. This approach remains the only way to obtain direct and adequate neural decompression as well as optimal spine reconstruction and fixation through a single surgical procedure [115]. *Katonis* et al. have recommended anterior decompression with corpectomy, reconstruction with a titanium cage filled with autograft, and stabilization with an anterior plate in cases of kyphotic deformity with cord compression caused by *Brucella* spondylitis in the lower thoracic spine, whereas when the infection was localized in the lumbar spine, a posterior approach and laminectomy were chosen [108]. *Yin* et al. more recently reported their results on treating 16 patients with lumbar *Brucella* spondylitis with one-stage anterior internal fixation, debridement, and bone fusion. The mean follow-up was 35.3 ± 8.1 months (range, 24–48 months). All patients were considered completely cured, with bone fusion achieved in 4.8 ± 1.3 months. Pain and neurological function were significantly improved between the preoperative and last follow-up visits, as well as kyphotic deformity, as the Cobb angle was 20.7 ± 9.8° preoperatively and measured 8.1 ± 1.3° at the last follow-up visit. The authors concluded that one-stage surgical treatment with anterior debridement, fusion, and instrumentation can be an effective and feasible treatment method for lumbar *Brucella* spondylitis [116].

However, the opponents of the anterior standalone approach in the treatment of vertebral osteomyelitis consider this approach inadequate to restore and to ensure stability of the infected spine and to correct kyphosis and, therefore, believe that supplemented posterior fixation is mandatory [117]. In order to determine the optimal surgical approach, *Na* et al. compared 2 groups of patients undergoing surgical treatment for lumbar *Brucella* spondylitis. The clinical and surgical outcomes were compared in terms of operative time, intraoperative blood loss, hospitalizations, bony fusion time, complications, visual analog scale score, recovery of neurological function, and deformity correction. Both anterior and posterior approaches were successful, and fusion was achieved within 11 months in all cases. Yet, the posterior approach resulted in better kyphotic deformity correction, less surgical invasiveness, and fewer complications [112]. Similar results have been reported by Jiang et al. in 62 patients with lumbar *Brucella* spondylitis who underwent either one-stage posterior pedicle fixation, debridement, and interbody fusion or anterior debridement, bone grafting, and posterior instrumentation. Both surgical interventions were equally effective in the treatment of lumbar *Brucella* spondylitis. However, the posterior approach demonstrates advantages such as reduced surgical time, less blood loss and hospital stays, and fewer perioperative complications. Therefore, the one-stage posterior pedicle fixation, debridement, and interbody fusion represent a superior treatment option [118]. Significant shorter operation time, hospitalization time, and intraoperative blood loss has also been reported in patients with thoracolumbar *Brucella* spondylitis who were treated with posterior debridement and instrumented fusion compared to patients treated with one-stage anterior radical debridement combined with bone grafting and fusion and posterior internal fixation (360° surgery). No significant difference has been found between the two groups in terms of pain control, neurological improvement and deformity correction [119].

#### 6.2.2. Minimally Invasive Surgical Techniques

Spinal brucellosis is less destructive compared to other infectious spinal diseases such as pyogenic spondylitis or spinal tuberculosis, and therefore, minimally invasive procedures should be preferred as much as possible in patients undergoing surgical treatment, especially in cases with poor general health and comorbidities [120]. Hadjipavlou et al. have reported their technique of percutaneous transpedicular discectomy and drainage of purulent material in patients with pyogenic spondylodiscitis [121,122], including patients with *Brucella* spondylitis [108]. In a series of 10 patients receiving surgical treatment for spinal brucellosis, 3 patients with spondylodiscitis without epidural abscesses underwent transpedicular discectomy and drainage with good and sustained results [108]. According to the authors this minimally invasive technique has high diagnostic and therapeutic effectiveness when applied in the early stages of uncomplicated spondylodiscitis because it promotes pain relief and healing by stimulating granulation tissue to enter the avascular disc space from the subchondral bone, but is contraindicated in the presence of instability, kyphosis from bone destruction, and neurological deficit [122]. This technique is also ideal for collecting samples for microbiological diagnosis with greater sensitivity compared to CT-guided biopsies [123].

Recently, *Wang* et al. retrospectively analyzed 13 patients with lumbar *Brucella* spondylitis who underwent bi-portal endoscopic decompression, debridement, and interbody fusion, combined with percutaneous screw fixation, with 92.3% of the patients reporting good to excellent outcomes [109]. Indications for this procedure are similar to those of open surgery and include severe disc or vertebral destruction resulting in intractable low back pain refractory to medication treatment, severe or progressive neurological dysfunction due to compression of the spinal cord or cauda equina by inflammatory tissue in the spinal canal or epidural abscesses, spinal instability, and ineffective medical therapy. However, this operation is contraindicated in cases with severe destruction of the anterior column requiring anterior debridement and interbody fusion through a retroperitoneal approach or in cases with massive paravertebral abscesses [124].

Other minimally invasive procedures, such as percutaneous endoscopic discectomy and drainage [125,126], percutaneous endoscopic debridement with dilute Betadine solution irrigation [123], and thoracoscopic debridement and stabilization [127], have been described for the management of bacterial spondylodiscitis, including cases of spinal tuberculosis, and could potentially be recruited for the surgical treatment of *Brucella* spondylitis.

## 7. Conclusions

Brucellosis is the most common zoonosis worldwide, posing a significant public health problem. Spinal involvement presenting as spondylitis, spondylodiscitis, and epidural, paravertebral, and psoas abscesses is a frequent and serious complication of the disease, often with post-treatment residual damage. The therapeutic approach of spinal brucellosis should always be multidisciplinary with a team of infectious disease specialists, microbiologists, radiologists, neurosurgeons, and orthopedics in order to achieve a favorable outcome.

## Data Availability

The data presented in this study are available on request from the corresponding author.
